# Supplementary material for: Getting More Out of Biomedical Documents with GATE's Full Lifecycle Open Source Text Analytics
Source: PLoS Comput Biol. 2013 Feb 7;9(2):e1002854. doi: 10.1371/journal.pcbi.1002854 (PMC3567135; doi:10.1371/journal.pcbi.1002854)
Supplement: Dataset S1 — GATE software. Dataset S1 bundled with this paper contains a distribution of GATE (or see http://gate.ac.uk/download/). (TGZ) [file pcbi.1002854.s001.tgz › plos-gate/src/gate/resources/texts/news/c&w-obs-13-May-2001.html]

Observer | C&W to spend £7bn cash pile on buying spree

**C&W to spend £7bn cash pile on buying spree**


**Heather Connon, investment editor**  
**Sunday May 13, 2001**  
**The Observer**

Cable & Wireless will commit itself this week to an aggressive acquisition strategy to use up its £7 billion cash pile. But it will not rule out giving some of the money back to shareholders in future.

Chief executive Graham Wallace has been under increasing pressure to return to investors some of the cash amassed from selling minority stakes and joint ventures around the world.

One of C&W's biggest institutional shareholders said £7bn was too much to spend: 'It is equal to half its market value. That is too much cash for their own good. They should give back £5bn.'

Others called on C&W to give them the cash to fund the £5.9bn rights issue launched by BT.

But Wallace is understood to believe it would be 'crazy' to give back the money when telecoms and internet businesses are trading at rock-bottom prices. Only £2bn is actually in its bank now. The rest - including a similar sum due from the sale of C&W Optus, the company's Australian division, to Singapore Telecom last March - is not yet in.

C&W's shares have halved in value since it issued a profit warning in March, and Wallace will be anxious to restore the company's credibility in the City.

He gained considerable credit for unravelling the network of shareholdings and joint ventures that made C&W more like an investment trust than a telecoms company, but the profit warning and the failure to make acquisitions have dented his reputation.

He will attempt to restore it by outlining a detailed acquisition strategy, showing how he will use the cash and explaining the level of debt he believes is optimum for the group. Although there have been talks about a number of deals, it is believed to be unlikely that any will come to fruition in time for Wednesday's results announcement.

C&W's name has been linked to virtually every telecoms business. Jim McCafferty, telecoms analyst with SG Warburg, said the acquisition of a US web-hosting business was likely to be a priority. The group's strategy has been to concentrate on internet protocol and business data. It has few interests in mobile phones, which have given its rivals problems.

But its disposal programme, coupled with the slowdown in its markets, means earnings this year - which it said in March would be between 14p and 16p - would not cover the dividend if it stays at last year's level of 16.5p. While the City does not expect a cut, which would send the wrong signal from a firm with so much cash, it could warn of a future reduction, with the compensation of a special dividend.

Guardian Unlimited © Guardian Newspapers Limited 2001
